# Kratom Alkaloids: Interactions With Enzymes, Receptors, and Cellular Barriers

**DOI:** 10.3389/fphar.2021.751656

**Published:** 2021-11-17

**Authors:** Nur Aziah Hanapi, Nelson Jeng-Yeou Chear, Juzaili Azizi, Siti R. Yusof

**Affiliations:** Centre for Drug Research, Universiti Sains Malaysia, Minden, Malaysia

**Keywords:** receptor-binding, mitragynine, *Mitragyna speciosa*, metabolism, kratom, alkaloids, drug-drug interactions, barrier permeability

## Abstract

Parallel to the growing use of kratom, there is a wealth of evidence from self-report, preclinical, and early clinical studies on therapeutic benefits of its alkaloids in particular for treating pain, managing substance use disorder, and coping with emotional or mental health conditions. On the other hand, there are also reports on potential health risks concerning kratom use. These two aspects are often discussed in reviews on kratom. Here, we aim to highlight specific areas that are of importance to give insights into the mechanistic of kratom alkaloids pharmacological actions. This includes their interactions with drug-metabolizing enzymes and predictions of clinical drug-drug interactions, receptor-binding properties, interactions with cellular barriers in regards to barrier permeability, involvement of membrane transporters, and alteration of barrier function when exposed to the alkaloids.

## 1 Introduction

**FIGURE 1:**
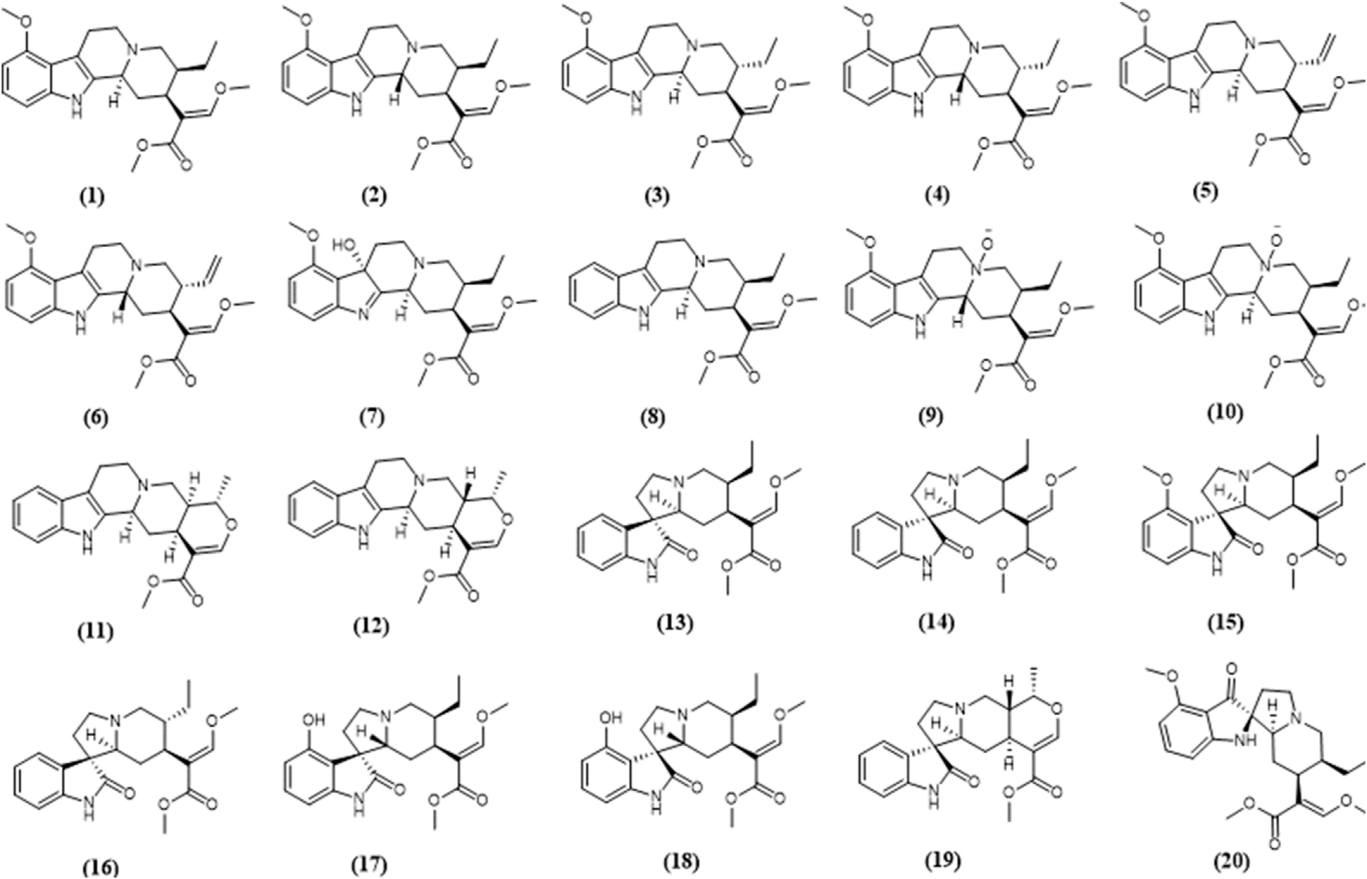
Chemical structures of selected kratom alkaloids—(**1**) mitragynine, (**2**) speciociliatine, (**3**) speciogynine, (**4**) mitraciliatine, (**5**) paynantheine, (**6**) isopaynantheine, (**7**) 7-hydroxymitragynine, (**8**) corynantheidine, (**9**) speciociliatine n-oxide, (**10**) mitragynine n-oxide, (**11**) tetrahydroalstonine, (**12**) ajmalicine, (**13**) corynoxine, (**14**) corynoxine B, (**15**) mitragynine oxindole B, (**16**) isorynchophylline, (**17**) isospeciofoline, (**18**) speciofoline, (**19**) mitraphylline, (**20**) mitragynine pseudoindoxyl.

Kratom (*Mitragyna speciosa* Korth.) use in the traditional settings in Southeast Asian countries particularly Malaysia and Thailand to treat minor ailments and to increase work endurance among manual laborers is not new. Reports on the use of kratom as a substitute for opium in Malaya have been published as early as in the 1930s (Burkill and Haniff, 1930; Burkill, 1935). Now, kratom use has spread to the West particularly in the United States of which kratom products are widely marketed online (Williams and Nikitin, 2020). Reasons for kratom use in the States include to self-treat acute and chronic pain, to reduce or abstain from using non-prescription opioids and/or heroin, and to a lesser extent as a substitute for the drugs, and to cope with emotional or mental health conditions such as anxiety, depression and post-traumatic stress disorder (Grundmann, 2017; Smith and Lawson 2017; Smith et al., 2021). The increasing use of kratom which is no longer limited to Southeast Asian countries has sparked many interests within the scientific community to investigate the therapeutic potential of the plant and possible health risks. A breadth of evidence is available on pharmacological actions of kratom preparations and alkaloids, primarily central actions of mitragynine and 7-hydroxymitragynine. Apart from the two most studied alkaloids, there is a growing number of other alkaloids being reported and to date, approximately 45 alkaloids were identified in kratom (Ramanathan et al., 2021). Chemical structures of kratom alkaloids which are discussed in the later sections of this review are shown in Figure 1. Findings from preclinical studies, for example, antinociceptive activity to some extent corroborated with data from self-report studies of which among the reasons for kratom use is to manage pain, further supported by the recent randomized controlled study in humans (Vicknasingam et al., 2020). This also seems to be the case for use of kratom to alleviate opioid withdrawal (Hassan et al., 2020) and to relieve anxiety (Hazim et al., 2014). Further investigations at the cellular and molecular level aid to gain an understanding of the mechanistic of kratom alkaloids actions.

Here, we highlight physiological interactions of kratom alkaloids focusing on interactions with enzymes, receptors, and cellular barriers. These are emerging areas of research concerning kratom alkaloids of which are of significant importance in determining potential development as therapeutics.

## 2 Interactions With Enzymes

### 2.1 Metabolism of Kratom Alkaloids

Metabolism facilitates the elimination of drugs from animals and humans through the conversion of the drugs to more water-soluble metabolites. There are two phases of drug metabolism i.e., phase I and phase II. Phase I metabolism includes hydrolysis, oxidation, and reduction reactions which are mainly catalyzed by various drug-metabolizing enzymes (DMEs). Phase II consists of a conjugation reaction involving glucuronidation and sulfation (Shapiro and Shear, 2001).

**FIGURE 2:**
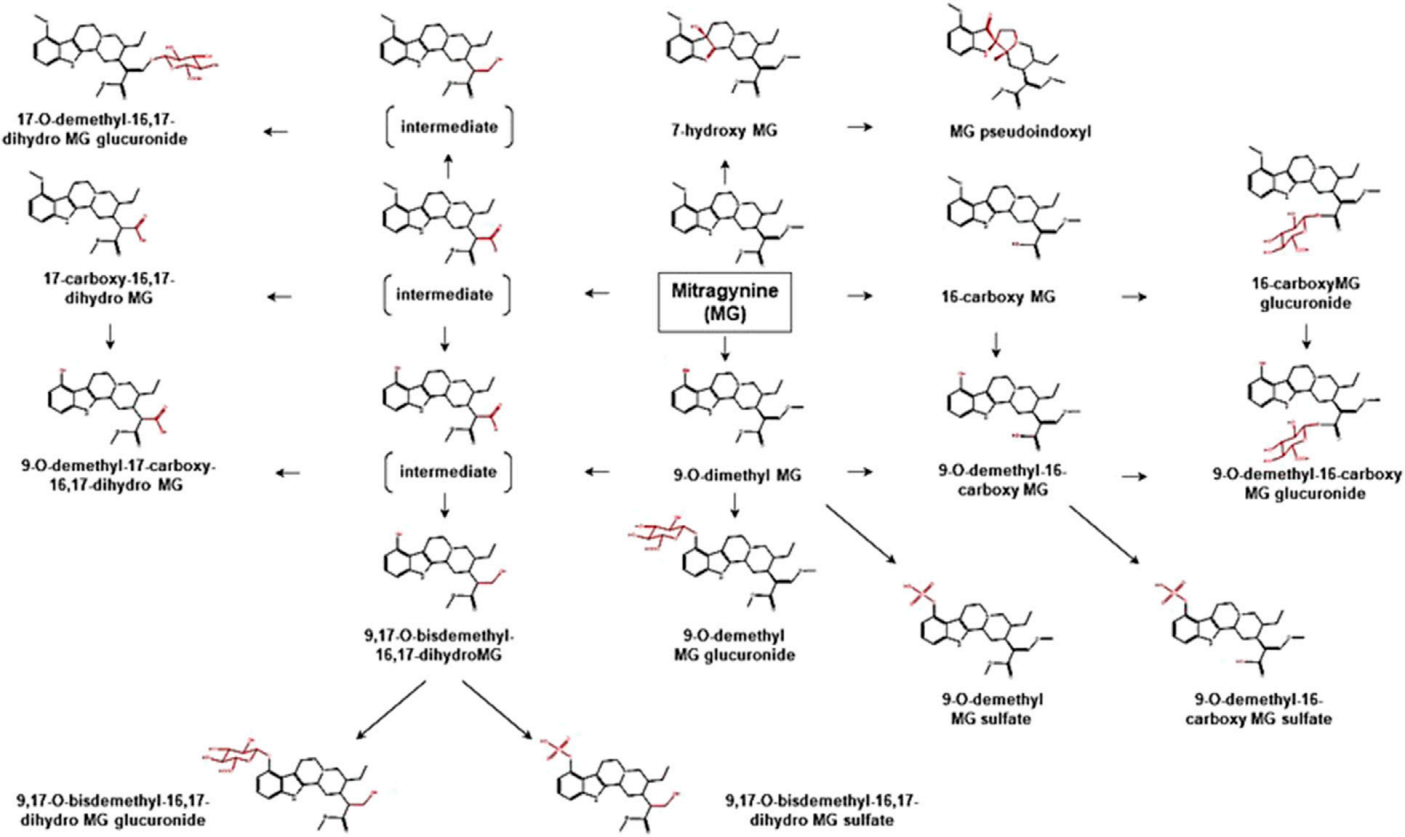
Proposed metabolic pathways of MG based on data obtained in rodents and humans. Structures highlighted in red denote structural transformation from parent molecule MG. Figure was modified from Philipp et al. (2009) and Kamble et al. (2020).

**FIGURE 3:**
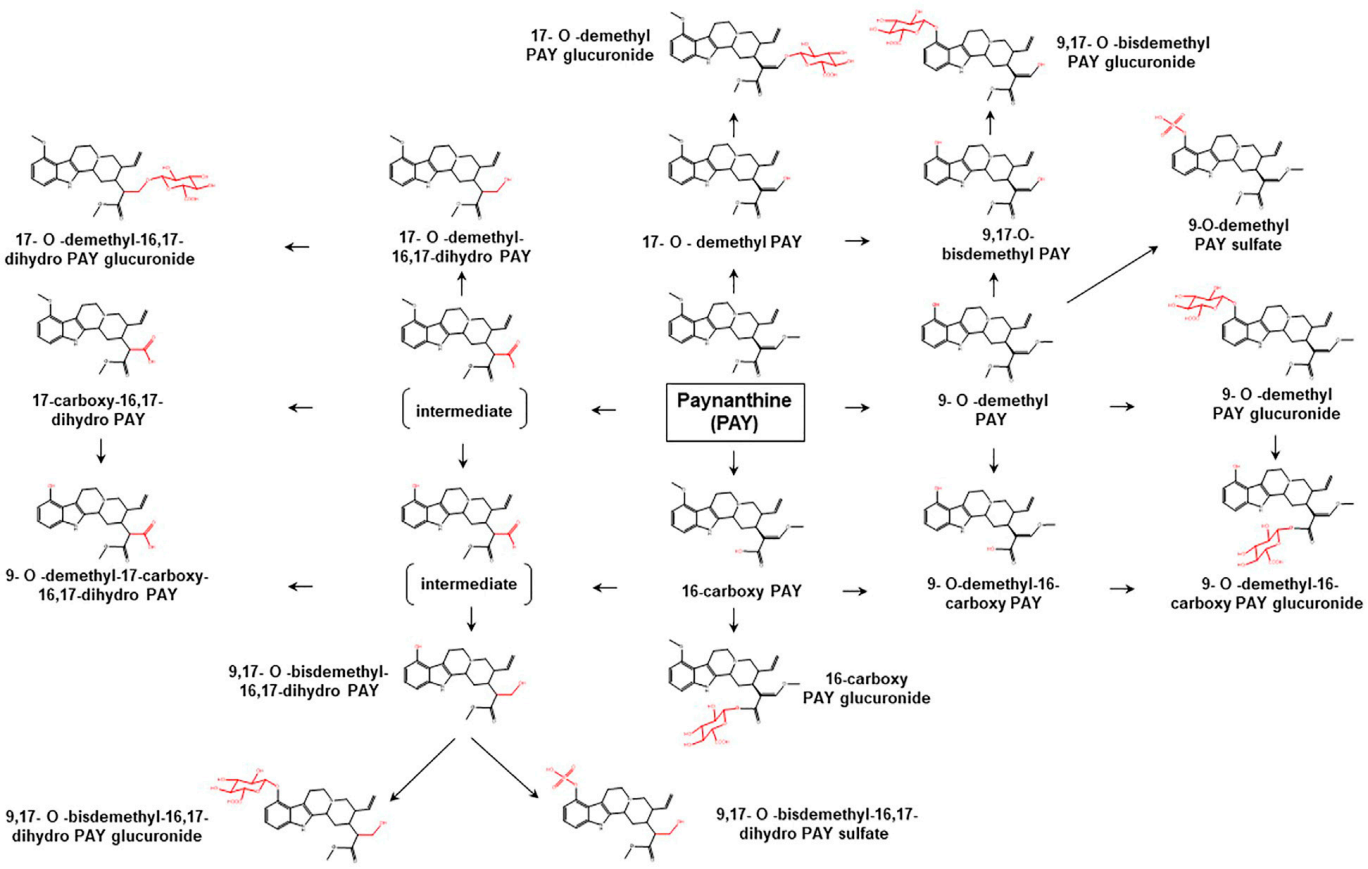
Proposed metabolic pathways of PAY based on data obtained in rodents and humans. Structures highlighted in red denote structural transformation from parent molecule PAY. Figure was modified from Philipp et al. (2010a).

**FIGURE 4:**
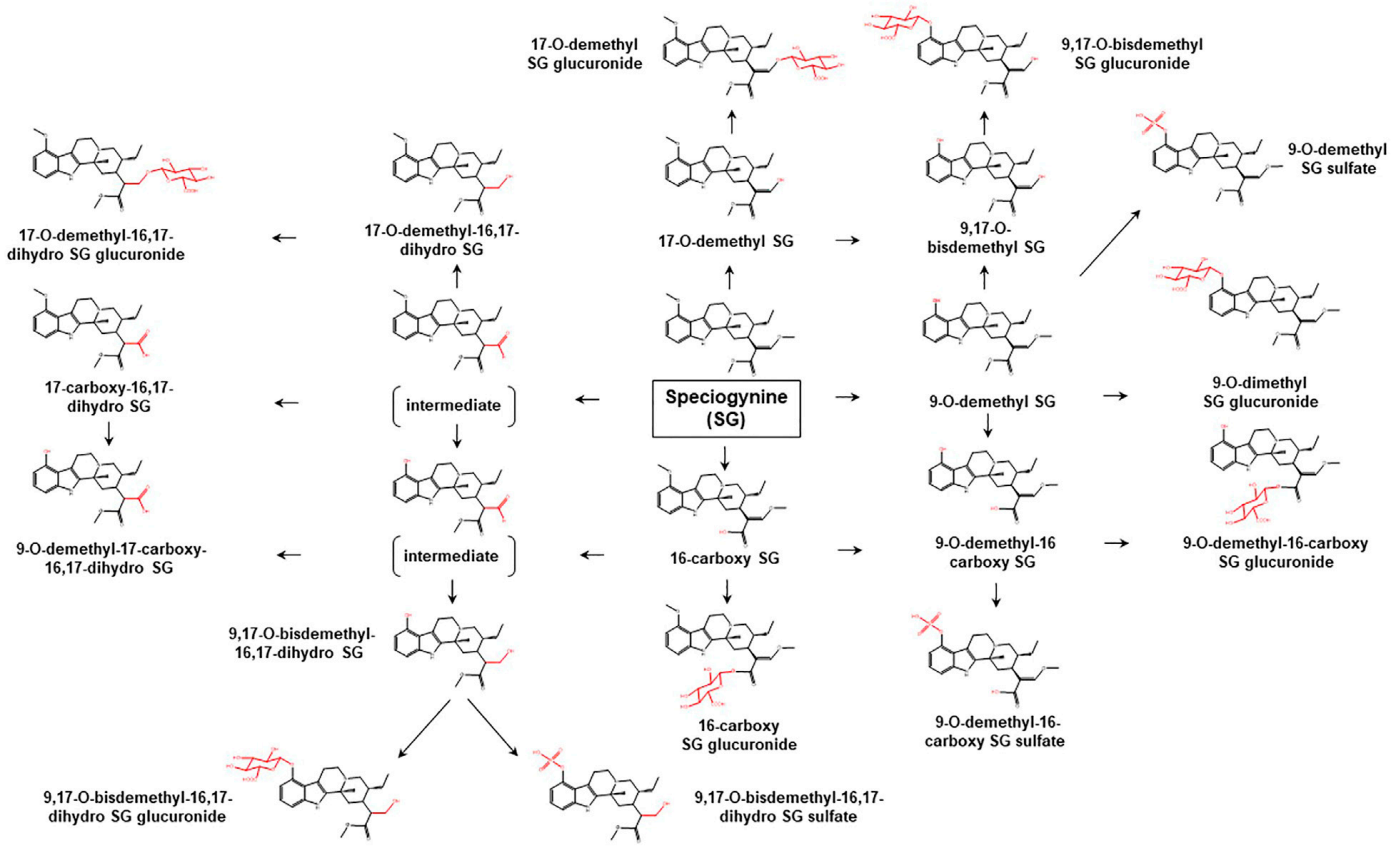
Proposed metabolic pathways of SG based on data obtained in rodents and humans. Structures highlighted in red denote structural transformation from parent molecule SG. Figure was modified from Philipp et al. (2010b).

**FIGURE 5:**
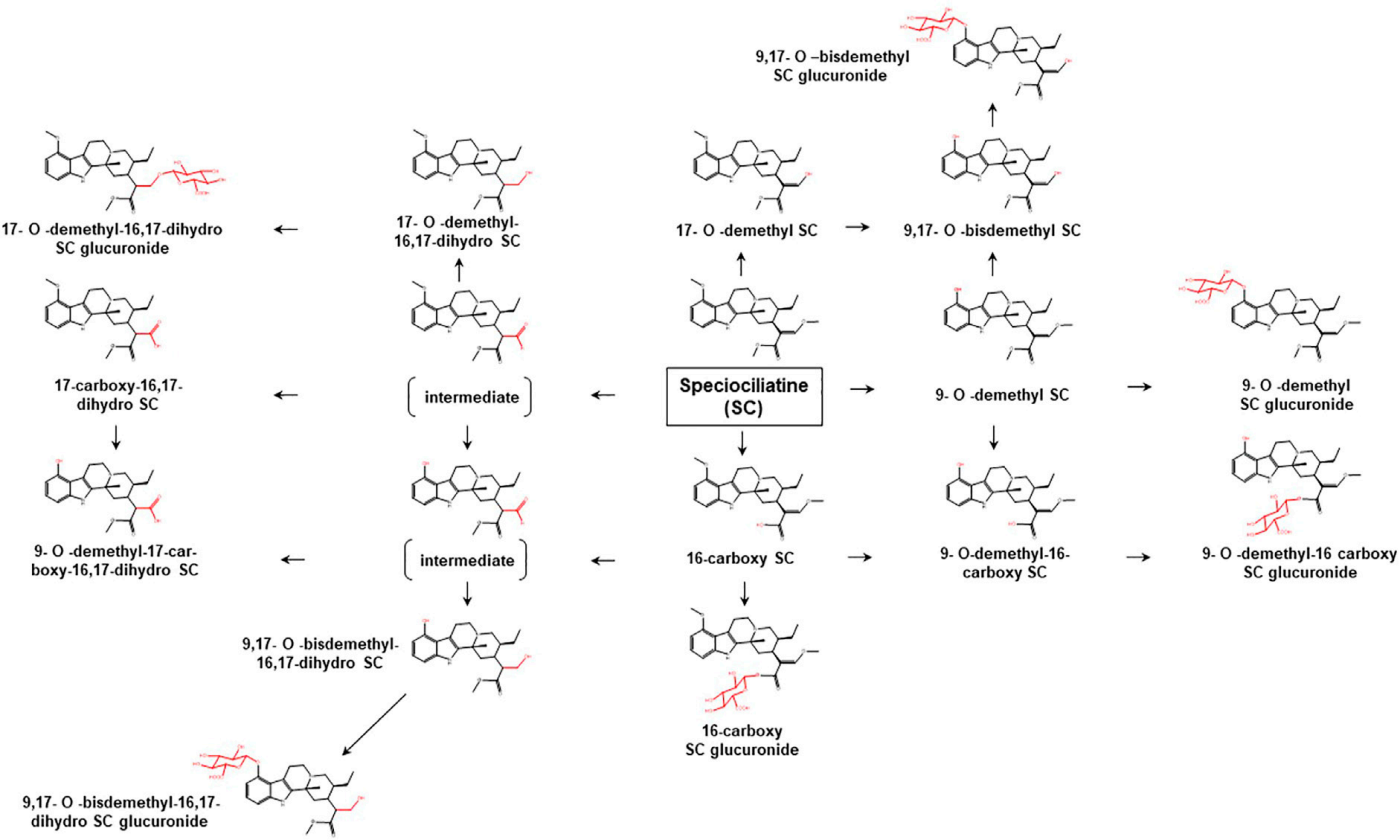
Proposed metabolic pathways of SC based on data obtained in rodents and humans. Structures highlighted in red denote structural transformation from parent molecule SC. Figure was modified from Philipp et al. (2011a).

**FIGURE 6:**
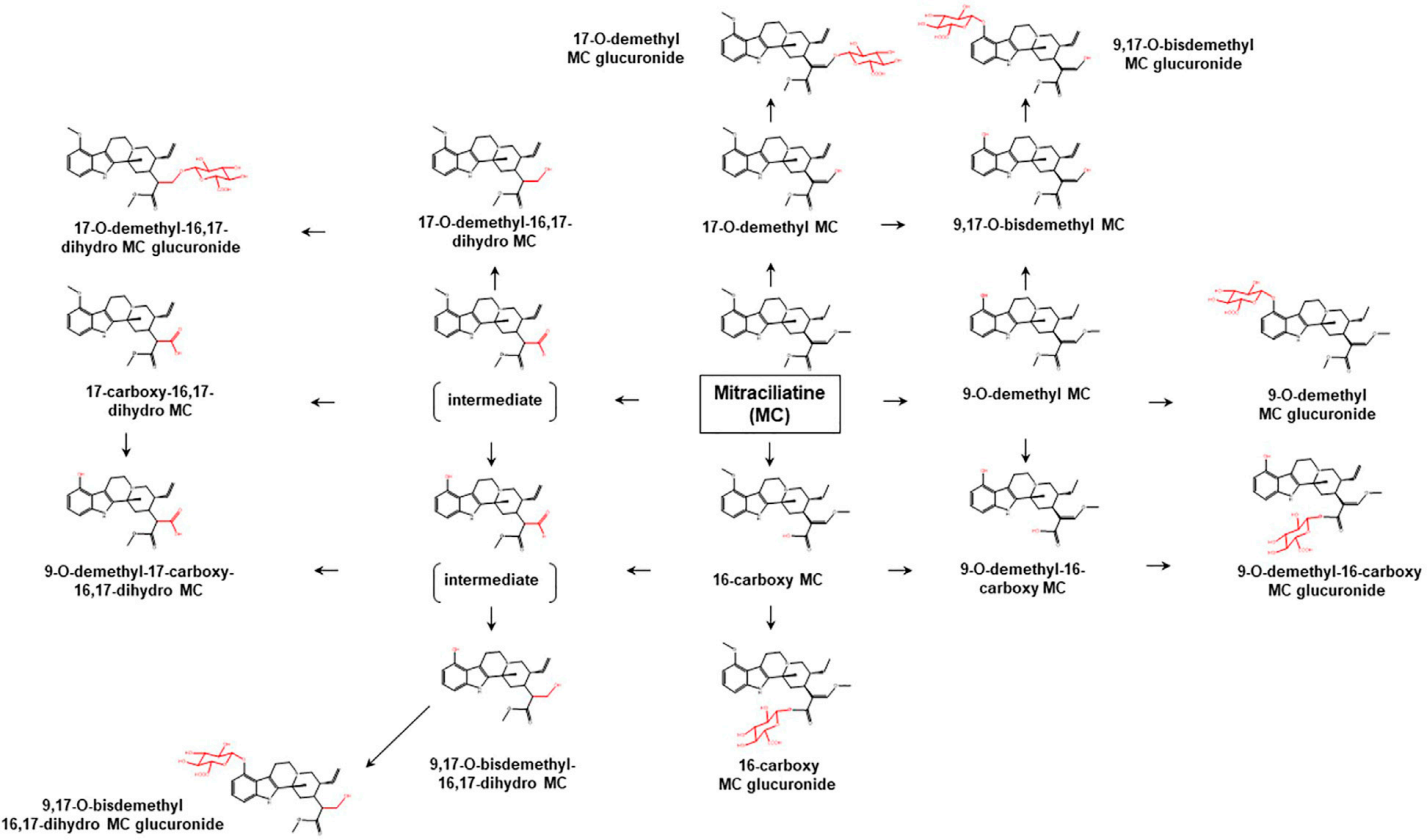
Proposed metabolic pathways of MC based on data obtained in rodents and humans. Structures highlighted in red denote structural transformation from parent molecule MC. Figure was modified from Philipp et al. (2011b).

**FIGURE 7:**
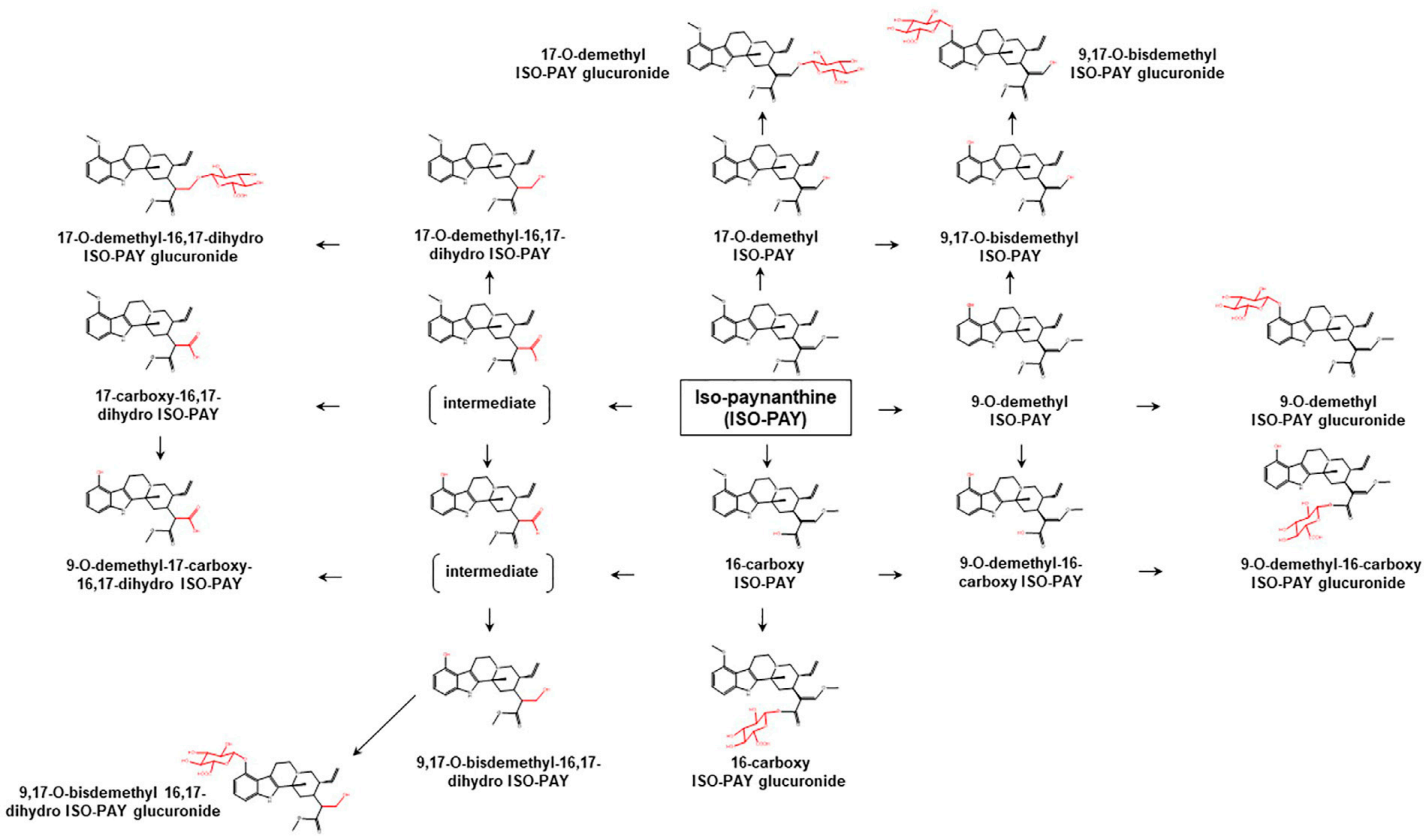
Proposed metabolic pathways of ISO-PAY based on data obtained in rodents and humans. Structures highlighted in red denote structural transformation from parent molecule ISO-PAY. Figure was modified from Philipp et al. (2011b).

To date, limited data are available concerning the metabolic pathways of kratom alkaloids and the involvement of various DMEs in the clearance of the alkaloids. Data from analyses of samples collected from rats and humans revealed that kratom alkaloids including mitragynine, speciogynine, paynantheine, speciociliatine, mitraciliatine, and isopaynantheine were extensively metabolized to multiple phase I and phase II metabolites. Phase I metabolism of the alkaloids involved hydrolysis of the methyl ester of the acrylic acid group at C-16, *O*-demethylation of the methoxy group at C-9 and C-17 positions, followed by oxidation to carboxylic acid or reduction to alcohol (Philipp et al., 2009; Philipp et al., 2010a; Philipp et al., 2010b; Philipp et al., 2011a; Philipp et al., 2011b). Following the phase I metabolism, some metabolites underwent phase II metabolism to produce glucuronide and sulfate conjugates (Phillip et al., 2009). The phase I and phase II metabolites of the alkaloids are tabulated in Table 1. In parallel to the list of metabolites, the proposed metabolic pathways for the alkaloids are illustrated in Figures 2–7.

**TABLE 1:** Phase I and II metabolites of kratom alkaloids in rat and human urine samples.

Alkaloid	Phase I metabolites		Phase II metabolites	
	Rat urine	Human urine	Rat urine	Human urine
MG^a^	1) 9-*O*-demethyl MG	1) 9-*O*-demethyl MG	Glucuronides of:	Glucuronides of:
	2) 16-carboxy MG	2) 16-carboxy MG	1) 9-*O*-demethyl MG	1) 9-*O*-demethyl MG
	3) 9-*O*-demethyl-16-carboxy MG	3) 17-*O*-demethyl-16,17-dihydro MG	2) 16-carboxy MG	2) 16-carboxy MG
	4) 17-*O*-demethyl-16,17-dihydro MG	4) 17-carboxy-16,17-dihydro MG	3) 9-*O*-demethyl-16-carboxy MG	3) 17-*O*-demethyl-16,17-dihydro MG
	5) 9,17-*O*-bisdemethyl-16,17-dihydro MG		4) 9,17-*O*-bisdemethyl-16,17-dihydro MG	
	6) 17-carboxy-16,17-dihydro MG			
	7) 9-*O*-demethyl-17-carboxy-16,17-dihydro MG			
			Sulfate of:	Sulfates of:
			1) 9-*O*-demethyl-16-carboxy MG	1) 9-*O*-demethyl MG
				2) 9-*O*-demethyl-16-carboxy MG
				3) 9,17-*O*-bisdemethyl-16,17-dihydro MG
PAY^b^	1) 9-*O*-demethyl PAY	1) 9-*O*-demethyl PAY	Glucuronides of:	Glucuronides of:
	2) 16-carboxy PAY	2) 16-carboxy PAY	1) 9-*O*-demethyl PAY	1) 9-*O*-demethyl PAY
	3) 9-*O*-demethyl-16-carboxy PAY	3) 17-carboxy-16,17-dihydro PAY	2) 16-carboxy PAY	2) 16-carboxy PAY
	4) 17-*O*-demethyl-16,17-dihydro PAY		3) 9-*O*-demethyl-16-carboxy PAY	
	5) 9,17-*O*-bisdemethyl-16,17-dihydro PAY		4) 17-*O*-demethyl-16,17-dihydro PAY	
	6) 17-carboxy-16,17-dihydro PAY		5) 9,17-*O*-bisdemethyl-16,17-dihydro PAY	
	7) 9-*O*-demethyl-17-carboxy-16,17-dihydro PAY		6) 17-*O*-demethyl PAY	
	8) 17-*O*-demethyl PAY		7) 9,17-*O*-bisdemethyl PAY	
	9) 9,17-*O*-bisdemethyl PAY			
			Sulfate of:	Sulfate of:
			1) 9,17-*O*-bisdemethyl-16,17-dihydro PAY	1) 9-*O*-demethyl PAY
SG^c^	1) 9-*O*-demethyl SG	1) 9-*O*-demethyl SG	Glucuronides of:	Glucuronides of:
	2) 16-carboxy SG	2) 16-carboxy SG	1) 9-*O*-demethyl SG	1) 9-*O*-demethyl SG
	3) 9-*O*-demethyl-16-carboxy SG	3) 17-carboxy-16,17-dihydro SG	2) 16-carboxy SG	2) 16-carboxy SG
	4) 17-*O*-demethyl-16,17-dihydro SG		3) 9-*O*-demethyl-16-carboxy SG	
	5) 9,17-*O*-bisdemethyl-16,17-dihydro SG		4) 17-*O*-demethyl-16,17-dihydro SG	
	6) 17-carboxy-16,17-dihydro SG		5) 9,17-*O*-bisdemethyl-16,17-dihydro SG	
	7) 9-*O*-demethyl-17-carboxy-16,17-dihydro SG		6) 17-*O*-demethyl SG	
	8) 17-*O*-demethyl SG		7) 9,17-*O*-bisdemethyl SG	
	9) 9,17-*O*-bisdemethyl SG			
			Sulfate of:	Sulfate of
			1) 9,17-*O*-bisdemethyl-16,17-dihydro SG	1) 9-*O*-demethyl SG
SC^d^	1) 9-*O*-demethyl SC	1) 9-*O*-demethyl SC	Glucuronides of:	Glucuronides of:
	2) 16-carboxy SC	2) 16-carboxy SC	1) 9-*O*-demethyl SC	1) 9-*O*-demethyl SC
	3) 9-*O*-demethyl-16-carboxy SC	3) 9-*O*-demethyl-16-carboxy SC	2) 16-carboxy SC	2) 16-carboxy SC
	4) 17-*O*-demethyl-16,17-dihydro SC		3) 9-*O*-demethyl-16-carboxy SC	3) 17-*O*-demethyl-16,17-dihydro SC
	5) 9,17-*O*-bisdemethyl-16,17-dihydro SC		4) 17-*O*-demethyl-16,17-dihydro SC	
	6) 17-carboxy-16,17-dihydro SC		5) 9,17-*O*-bisdemethyl-16,17-dihydro SC	
	7) 9-*O*-demethyl-17-carboxy-16,17-dihydro SC		6) 9,17-*O*-bisdemethyl SC	
	8) 17-*O*-demethyl SC			
	9) 9,17-*O*-bisdemethyl SC			
MC^e^	1) 9-*O*-demethyl MC	1) 9-*O*-demethyl MC	Glucuronides of	Glucuronide of:
	2) 16-carboxy MC		1) 9-*O*-demethyl MC	1) 9-*O*-demethyl MC
	3) 9-*O*-demethyl-16-carboxy MC		2) 16-carboxy MC	
	4) 17-*O*-demethyl-16,17-dihydro MC		3) 9-*O*-demethyl-16-carboxy MC	
	5) 9,17-*O*-bisdemethyl-16,17-dihydro MC		4) 17-*O*-demethyl-16,17-dihydro MC	
	6) 17-carboxy-16,17- dihydro MC		5) 9,17-*O*-bisdemethyl-16,17-dihydro MC	
	7) 9-*O*-demethyl-17-carboxy-16,17-dihydro MC		6) 17-*O*-demethyl MC	
	8) 17-*O*-demethyl MC		7) 9,17-*O*-bisdemethyl MC	
	9) 9,17-*O*-bisdemethyl MC			
ISO-PAY^e^	1) 9-*O*-demethyl ISO-PAY	1) 9-*O*-demethyl ISO-PAY	Glucuronides of:	
	2) 16-carboxy ISO-PAY	2) 17-carboxy-16,17-dihydro ISO-PAY	1) 9-*O*-demethyl ISO-PAY	
	3) 9-*O*-demethyl-16-carboxy ISO-PAY		2) 16-carboxy ISO-PAY	
	4) 17-*O*-demethyl-16,17-dihydro ISO-PAY		3) 17-*O*-demethyl-16,17-dihydro ISO-PAY	
	5) 9,17-*O*-bisdemethyl-16,17-dihydro ISO-PAY		4) 17-*O*-demethyl ISO-PAY	
	6) 17-carboxy-16,17-dihydro ISO-PAY			
	7) 9-*O*-demethyl-17-carboxy-16,17-dihydro ISO-PAY			
	8) 17-*O*-demethyl ISO-PAY			
	9) 9,17-*O*-bisdemethyl ISO-PAY			

MG, mitragynine; PAY, paynantheine; SG, speciogynine; SC, speciociliatine; MC, mitraciliatine; ISO-PAY, isopaynantheine.

a Phillip et al.(2009);

b Phillip et al. (2010a);

c Phillip et al. (2010b);

d Phillip et al. (2011a);

e Phillip et al. (2011b).

Kamble et al. (2019) characterized the metabolic profile of mitragynine against various cytochrome P450 (CYP)-containing systems including human liver microsomes (HLM), human liver S9 (HLS9), and recombinant CYP enzymes. In the HLM system, four oxidative species including 7-hydroxymitragynine and one *O*-demethylated metabolite i.e., 9-*O*-demethylmitragynine were detected as the prevalent metabolites of mitragynine, in accord with Basiliere and Kerrigan (2020a). None of the mitragynine phase I metabolites was discovered to be conjugated with glutathione. The metabolite profiling of mitragynine was comparable in HLM and HLS9, where both systems demonstrated a minor metabolic pathway. On the other hand, CYP3A4 was discovered as the major CYP isoform responsible for the metabolism of mitragynine with small or negligible contributions from CYP2C9, 2C19, and 2D6. The data on metabolic pathways of mitragynine via recombinant CYP enzymes were further evaluated against a series of multiple isoforms of 1A2, 2B6, 2C8, 2C9, 2C18, 2C19, 2D6, and 3A4 (Basiliere and Kerrigan, 2020b). Only 2C18, 2C19, 2D6, and 3A4 isoforms displayed metabolic activities among the tested enzymes. The results indicate that 9-*O*-demethylmitragynine was the most abundant metabolite produced by CYP2C19, 2D6, and 3A4, while 9-*O*-demethyl-16-carboxymitragynine was the least prevalent metabolite hydrolyzed by 2C19. 16-Carboxymitragynine was produced by CYP2C19 and 2D6 while 7-hydroxymitragynine was only produced by CYP3A4. Although data from Kamble et al. (2019) showed negligible metabolic activity expressed by CYP2C19 and 2D6, the data reported by Basiliere and Kerrigan (2020b) indicate that both isoforms were capable of metabolizing mitragynine to several important metabolites.

### 2.2 Potential Drug-Drug Interactions

This section deals with interactions of mitragynine and related alkaloids in modulating enzymes especially for enzymes that pose clinical importance. As the DMEs are the primary route of drug clearance in the human body (Di, 2014), modulation of the expression or function of DMEs through inhibition or induction by one or more chemicals that affect the metabolism of clinical drugs may lead to toxic effects or lack of clinical efficacy (Food and Drug Administration Center for Drug Evaluation and Research, 2020). The serious impact of DMEs modulation by other chemicals leads to the universal use of the term drug-drug interaction (DDI) to specifically refer to this type of interaction. DDI is often the primary obstacle in drug discovery and development and causes many clinically approved drugs to be withdrawn from the market (Wienkers and Heath, 2005). DDI can be recognized earlier in the preclinical phase by good experimental designs and by following guidelines provided by regulating agencies such as the U.S. Food and Drug Administration (FDA) (Food and Drug Administration Center for Drug Evaluation and Research, 2020) and European Medicines Agency (European Medicines Agency, 2012). Interactions of mitragynine with enzymes other than DMEs have also been reported. However, studies on these enzymes were very limited, with just acetylcholinesterase (Innok et al., 2021) and cyclooxygenase (Utar et al., 2011) being evaluated. Findings from these studies were less clinically relevant as the experiments were performed using enzymes from non-human sources i.e., electric eel for acetylcholinesterase, and rodent macrophage cell lines for cyclooxygenase, or the inhibition data showed mitragynine concentration that is hardly attained in human plasma, for example, IC_50_ of 264 µM for acetylcholinesterase.

It is obvious from the preceding section that mitragynine and other related alkaloids are substrates for multiple CYP isoforms and hence may interfere with metabolisms of clinical drugs. Preclinical research on mitragynine and related alkaloids on DDI is limited but has been gaining attention within the last 10 years (Hanapi et al., 2013; Lim et al., 2013; Kamble et al., 2020; Todd et al., 2020; Tanna et al., 2021). Here, the focus is on the effect of mitragynine and related alkaloids on DMEs from *in vitro* preclinical research, and their utility to predict clinical DDI. Only studies on human DMEs that make clinical prediction possible were included in this review (Table 2).

**TABLE 2:** Extent of mitragynine and related alkaloids inhibition on major human drug metabolizing enzymes (DMEs).

References	Enzyme system	CYPs isoform	Alkaloids	Key findings	Prediction to clinical DDI is possible?	Following FDA/EMA guideline?
Tanna et al. (2021)	Human liver microsomes; human intestinal microsomes	2C19, 2D6, 3A	Mitragynine	Mitragynine is a competitive inhibitor for CYP2D6 (K_i_ = 1.17 µM)	Yes	Yes
				Mitragynine is a mechanism-based inhibitor for CYP3A4 (HLM: K_I_ = 4.1 µM, K_inact_ = 0.068 min^−1^; HIM: K_I_ = 4.2 µM, K_inact_ = 0.079 min^−1^)		
Todd et al. (2020)	Human liver microsomes	2C9, 2D6, 3A	Mitragynine, 7-hydroxymitragynine, and speciofoline	Mitragynine at 100 μM inhibit >80% for CYP2C9, CYP2D6, and CYP3A	No	No
				7-Hydroxymitragynine at 100 μM inhibit >80% for CYP2D6		
				Speciofoline at 100 μM inhibit >80% for CYP2C9 and CYP3A		
Kamble et al. (2020)	Human liver microsomes	1A2, 2C8, 2C9, 2C19, 2D6, 3A4/5	Mitragynine, speciogynine,	Mitragynine and corynantheidiene is a competitive inhibitor for CYP2D6 activity with K_i_ values of 1.1 and 2.8 µM respectively	Yes	Yes
			speciociliatine, corynantheidine, 7-hydroxymitragynine, and paynantheine			
Hanapi et al. (2013)	Baculovirus hCYP450	1A2, 2D6, 3A4	Mitragynine	Mitragynine is a non-competitive inhibitor for CYP2C9 (K_i_ = 61.48 µM) and CYP2D6 (K_i_ = 12.86 µM)	Yes	No
	Expression system (baculosomes); human liver cancer cell line (HepG2)			Mitragynine is a competitive inhibitor for CYP3A4 (K_i_ = 379.18 µM)		
Lim et al. (2013)	Baculovirus hCYP450	2C9, 2D6, 3A4	Mitragynine	Mitragynine inhibit CYP3A4 with IC_50_ value 3.98 μM (testosterone) and 17.3 μM (midazolam)	Yes	No
	Expression system (baculosomes)			Mitragynine induce CYP1A2 mRNA and protein expression as well as enzyme activity		

HLM, human liver microsomes; HIM, human intestinal microsomes; K_i_ reversible inhibition constant; K_I_, time-dependent inhibition constant; K_inact_ maximum rate of inactivation.

The CYPs 1A2, 2C9, 2C19, 2D6, 2E1, and 3A4 isoforms are the major phase I DMEs responsible for the metabolism of over 90% of commercially marketed drugs (Lynch and Price, 2007; Bibi, 2008). The first study of mitragynine interactions with CYPs by Hanapi et al. (2013) set precedence to the subsequent studies that revealed more detailed information on the mechanisms and strength of DDI. The inhibitory constant parameter such as IC_50_ and K_i_ gathered from these studies were useful to support the prediction of potential clinical DDI through a static mechanistic model or physiologically based pharmacokinetic modeling (Obach et al., 2006; Templeton et al., 2016; Tod et al., 2016). For example, using the static mechanistic model, a ratio of the area under the plasma versus concentration-time curve (AUCR) for known CYP isoform substrate in the presence to absence of inhibitor could be estimated. An AUCR > 1.25 may suggest potential clinical DDI (Food and Drug Administration Center for Drug Evaluation and Research, 2020). The cut-off point of K_i_ < 12 µM to group mitragynine and other alkaloids as a potential clinical inhibitor of DMEs in this review was based on the guidelines provided by Tanna et al. (2021). Mitragynine K_i_ value from DMEs inhibition study <12 µM denote potential clinical relevance of CYP inhibition, determined relative to the highest mitragynine concentration quantified from autopsy blood samples of kratom-related death (Gershman et al., 2019; Tanna et al., 2021). For enzyme induction, a recent FDA guideline was used to classify CYP induction as potentially clinically significant (Food and Drug Administration Center for Drug Evaluation and Research, 2020). According to the guideline, a drug is interpreted as an inducer if the fold change of CYP mRNA expression relative to the vehicle control is ≥2-fold at the expected hepatic concentrations of the drug and/or if the increase is >20% of the response of the positive control *in vitro* cell-based assay.

Mitragynine and other alkaloids inhibited the *O*-deethylation reaction of CYP1A2 substrate phenacetin with a K_i_ value greater than the concentration that could be obtained in human plasma (Kamble et al., 2020). However, a study with human liver cancer cell line HepG2 showed the imminent potential of CYP1A2 induction by mitragynine (Lim et al., 2013). The mRNA expression for CYP1A2 when exposed to 10 µM mitragynine exceeded 2-fold relative to negative control and the increase was approximately 28% of the response of the CYP1A2 known inducer omeprazole in the cell-based assay (Lim et al., 2013). For the CYP2C subfamily, mitragynine and related alkaloids did not appreciably inhibit CYP2C8 and CYP2C9 (Kamble et al., 2020). However, mitragynine and speciociliatine inhibited CYP2C19 catalyzed S-mephenytoin hydroxylation with IC_50_ values of 10.6 and 8 µM respectively (Kamble et al., 2020). Although the study by Kamble et al. (2020) did not measure K_i_ for mitragynine and speciociliatine, estimation through Cheng-Prusoff equation (Yung-Chi and Prusoff, 1973; Haupt et al., 2015) for competitive inhibitor (K_i_ = IC_50_/2) may suggest potential clinical DDI between mitragynine (K_i_ ∼ 5 µM) and speciociliatine (K_i_ ∼ 4 µM) for drugs mainly metabolized by CYP2C19. Speciociliatine was the third [0.29% (w/w)] major alkaloid after mitragynine [3.8% (w/w)] in the Malaysian strain of kratom juice preparation and may significantly reach the K_i_ concentration in chronic kratom users assuming the intestinal absorption similar to mitragynine (Singh et al., 2020a).

Mitragynine has been repeatedly shown in different *in vitro* studies to potently inhibit CYP2D6 with K_i_ values ranging from 1.1 to 13 µM (Hanapi et al., 2013; Kamble et al., 2020; Todd et al., 2020; Tanna et al., 2021). Using the static mechanistic model, Tanna et al. (2021) revealed that kratom preparation sold in the U.S. market could cause significant DDI with drugs primarily metabolized by CYP2D6 if more than 9 g kratom extract containing 83 mg mitragynine (Todd et al., 2020) was taken with AUCR > 1.25. Based on this finding, the reported daily intake average i.e., 2.7 glasses of traditionally prepared kratom juice among chronic kratom users in Malaysia (Singh et al., 2020a) is sufficient to cause significant DDI with dextromethorphan (AUCR ∼ 1.4). Mitragynine did not appear to have a significant effect on the mRNA expression of CYP2D6. Although there was a substantial protein induction based on a qualitative technique for protein expression, the fold-induction did not qualify mitragynine as a clinically relevant CYP2D6 inducer (Lim et al., 2013).

Mitragynine was initially thought not to effectively inhibit the CYP3A4 isoform in a bioluminescent experiment with an IC_50_ of 41.32 µM (Hanapi et al., 2013). Subsequent studies with HLM support the previous data but with a much lower IC_50_ of < 20 µM when FDA recommended CYP3A4 probe substrate midazolam was used (Kamble et al., 2020; Tanna et al., 2021). Mitragynine also appears to inhibit CYP3A4 catalyzed midazolam hydroxylation in human intestinal microsomes (HIM) with IC_50_ = 21.9 µM (Tanna et al., 2021). Interestingly, mitragynine IC_50_ for CYP3A4 reduced substantially to 2.6 µM (HLM) and 3.2 µM (HIM) in a time-dependent inhibition experimental design (Tanna et al., 2021). The time-dependent inhibition observed from the study highlighted a mechanism-based inhibition that was irreversible and more potent for CYP3A4. In this type of inhibition, a product of mitragynine metabolism is covalently bound to the CYP3A4 active site instead of being released, which rendered the enzyme unavailable for other reactions (Deodhar et al., 2020). This observation was frequently missed in classical IC_50_ assays as the study design limit sufficient formation of active metabolites to form and deactivate the CYP. The impact of this finding is huge as roughly 40% of clinical drugs are substrates for CYP3A4 metabolism (Schaffenburg et al., 2021). The static mechanistic model demonstrated that as little as 2 g kratom powder containing 21 mg mitragynine (Todd et al., 2020) will precipitate DDI with CYP3A4 substrate midazolam (AUCR = 5.7) (Tanna et al., 2021). Similarly, about half-glass (∼150 ml) of traditionally prepared Malaysian kratom juice daily (Singh et al., 2020a) would be estimated to progressively increase the plasma level of midazolam by ∼ 6 fold. The influence of mitragynine on midazolam clearance would be substantially greater among chronic kratom users in Malaysia with AUCR > 12. On the other hand, the effects of mitragynine on the CYP3A4 mRNA, protein, and enzymatic activity in HepG2 cells were all below the criteria to suggest a significant *in vitro* induction effect (Lim et al., 2013).

## 3 Interactions of Kratom Alkaloids With Central Nervous System Receptors

The effects of kratom alkaloids on central nervous system (CNS) receptors have been extensively studied *in vitro* and *in vivo* assays. *In vitro* radioligand binding studies revealed that kratom alkaloids interact with opioid μ, δ, κ subtypes, and non-opioid receptors including alpha-1A, alpha-2A, 5-HT1A, 5-HT2A, D1, and D2 (Takayama et al., 2002; Boyer et al., 2008; Kruegel et al., 2016; Ellis et al., 2020; Obeng et al., 2020; Chear et al., 2021; Obeng et al., 2021). *In vivo* studies demonstrated that kratom alkaloids exert central analgesic, anti-anxiety, anti-drug addiction, and antipsychotic effects primarily through activation of central opioidergic, adrenergic, serotoninergic, and dopaminergic neurotransmission systems (Matsumoto et al., 1996a; Matsumoto et al., 1996b; Matsumoto et al., 1997; Takayama et al., 2002; Hazim et al., 2014; Vijeepallam et al., 2016; Foss et al., 2020; Obeng et al., 2020; Chear et al., 2021; Obeng et al., 2021). To better understand the CNS pharmacological targets of kratom alkaloids, this section is structured as follows: opioid receptors and non-opioid receptors (adrenergic, serotonin, and dopamine receptors).

### 3.1 Opioid Receptors

Kratom extracts (alcoholic, water, and alkaloid-enriched extracts) and the main alkaloid i.e., mitragynine demonstrated significant central analgesic activity in rodents and humans, and were fully antagonized by the non-selective opioid antagonists such as naloxone or naltrexone in most cases (Matsumoto et al., 1996a; Shaik Mossadeq et al., 2009; Sabetghadam et al., 2010; Carpenter et al., 2016; Vicknasingam et al., 2020). These suggest that the central analgesic effects of kratom and mitragynine are primarily mediated by opioid receptors (Ramanathan et al., 2021). Takayama et al. (2002) were the first to report on the interaction of mitragynine and its related indole alkaloids, 7-hydroxymitragynine, corynantheidine, and speciociliatine with μ-opioid receptors derived from guinea pig (Takayama et al., 2002). The opioid agonistic activity of the alkaloids was evaluated *ex vivo* by measuring electrically-induced twitch contraction in guinea pig ileum. Receptor binding affinities of the alkaloids at guinea pig μ, κ, and δ opioid receptors were determined by radioligand displacement assay against [^3^H]DAMGO, [^3^H]DPDPE, and [^3^H]U69593 respectively. In guinea pig ileum, mitragynine inhibited the electrically-induced twitch contraction with a pD2 value of 6.59, which was reversed by naloxone (300 nM). The pD2 or also known as pEC_50_ is the negative logarithm to base 10 of the EC_50_ of an agonist which indicates the potency but not the efficacy of the agonist. This suggests that mitragynine acted as an opioid agonist, but one that is weaker than morphine (pD2 = 7.17). Both the oxidized mitragynine i.e., 7-hydroxymitragynine and mitragynine pseudoindoxyl showed greater opioid agonistic activity than their precursor with pD2 values of 8.20 and 8.71 respectively. The two alkaloids were also more potent than morphine with relative potencies of 1,071 and 3,467%. Relative potency is defined as a percentage of the pD2 value of the tested compound against the reference drug, in this case, morphine. Mitragynine, 7-hydroxymitragynine, and mitragynine pseudoindoxyl showed selective binding affinities to the μ-opioid receptor in the radioligand binding assay against [^3^H]DAMGO, indicating that the alkaloids were μ-opioid receptor agonists. Speciociliatine on the other hand was found to weakly inhibit the twitch contraction with a relative potency of 2% (pD2 = 5.40). Corynantheidine (9-demethylated mitragynine) did not show opioid agonistic activity in the guinea pig ileum model. However, corynantheidine was later discovered to inhibit morphine-induced twitch contraction in guinea pig ileum with selective binding affinity to the μ-opioid receptor. This finding suggests that corynantheidine is a functional and selective μ-opioid receptor antagonist. Based on the above findings, it could be postulated that 1) *S*-orientation at the C-3 position of mitragynine is important for opioid-agonistic activity; 2) oxidation at indole B-ring enhances the opioid-agonistic activity; 3) the loss of Nb lone pair at C-ring abolishes the opioid agonistic activity; 4) the loss of 9-methoxy group abolishes the opioid-agonistic activity.

For the past 5 years, the interactions of kratom alkaloids with human opioid receptors have been extensively studied using various *in vitro* and *in vivo* assays. Kruegel et al. investigated binding affinity and functionality of mitragynine, 7-hydroxymitragynine, speciociliatine, speciogynine and paynantheine at human µ (hMOR), δ (hDOR) and ĸ (hKOR) opioid receptors using radioligand displacement and bioluminescence resonance energy transfer (BRET) functional assays (Kruegel et al., 2016). In general, the indole-based kratom alkaloids showed greater binding affinities at hMOR and hKOR with K_i_ values in submicromolar and micromolar ranges compared to hDOR (K_i_ > 10 μM). Among the tested alkaloids, 7-hydroxymitragynine had the highest and selective affinity for hMOR with a K_i_ value of 47 nM, followed by mitragynine (K_i_ = 233 nM), paynantheine (K_i_ = 410 nM), speciociliatine (K_i_ = 560 nM), and speciogynine (K_i_ = 728 nM). In addition, 7-hydroxymitragynine was also bound to hKOR and hDOR with K_i_ values of 188 and 219 nM respectively. In the BRET assay, mitragynine and 7-hydroxymitragynine showed potent agonistic activity at hMOR with EC_50_ values of 339 and 34.5 nM respectively. The two alkaloids acted as partial agonists at hMOR with maximal efficacy, E_max_ of 34 and 47% respectively, when compared to the full agonist DAMGO in antagonist experiments. In contrast, at hKOR, mitragynine and 7-hydroxymitragynine acted as competitive antagonists with IC_50_ values of 8.5 and 7.9 μM, and pA2 values of 1.4 and 0.49 μM respectively, when compared to the reference agonist U-50488. The pA2 reflects the affinity of an antagonist to a receptor. The value of pA2 is a negative logarithm of the molar concentration of the competitive antagonist, implying that the agonist concentration must be doubled to compensate for the antagonist’s action. Paynantheine, speciogynine, and speciocilatine showed weak competitive antagonist activities at both hMOR and hKOR with EC_50_ or IC_50_ values in micromolar ranges. Interestingly, all tested kratom alkaloids were also reported as competitive antagonists at mouse MOR, indicating the possibility of intra-species variation between the *in vitro* functional assays. Later in 2020, Obeng et al. also reported the opioid-like activity of selected indole-based kratom alkaloids i.e., 7-hydroxymitragynine, mitragynine, speciociliatine, and corynantheidine using radioligand displacement and homogenous time-resolved fluorescence (HTRF) assays (Obeng et al., 2020). In the study, 7-hydroxymitragynine was predominantly bound to hMOR (Ki = 7.16 nM), followed by hKOR (K_i_ = 74.1 nM) and hDOR (K_i_ = 236 nM). The binding strength of kratom alkaloids at hMOR was reported as follows: 7-hydroxymitragynine (K_i_ = 7.16 nM) > speciociliatine (K_i_ = 54.5 nM) > corynantheidine (K_i_ = 118 nM) > mitragynine (K_i_ = 161 nM). Similarly, 7-hydroxymitragynine also exhibited the highest binding affinity to hKOR with a K_i_ value of 74.1 nM, followed by speciociliatine (K_i_ = 116 nM), mitragynine (K_i_ = 198 nM), and corynantheidine (K_i_ = 1910 nM). In the HTRF assay, 7-hydroxymitragynine acted as a full agonist at hMOR (EC_50_ = 7.6 nM), and competitive antagonist at both hKOR and hDOR. Both mitragynine and speciociliatine were partial agonists at hMOR with EC_50_ values of 307.5 and 39.2 nM respectively. The *in vivo* opioid agonistic activities of 7-hydroxymitragynine, speciociliatine, and mitragynine were then evaluated using the hot-plate test in rats. Speciociliatine produced antinociceptive response with an ED_50_ value of 6.25 mg/kg, which was comparable to morphine (ED_50_ = 5.10 mg/kg) but weaker than 7-hydroxymitragynine (ED_50_ = 1.91 mg/kg). Similar to the *in vitro* hMOR binding and functional studies, mitragynine also exhibited the weakest antinociceptive effect (E_max_ 17.3%) among the tested alkaloids at the highest dose tested (10 mg/kg, i.v.). The antinociceptive action of speciociliatine, 7-hydroxymitragynine, mitragynine, and morphine were fully antagonized by naltrexone (0.1 mg/kg, i.v.). In the study, speciociliatine demonstrated opioid agonistic activity at hMOR, which is in contrast with findings reported by Kruegel et al. (2016) where the compound showed weak antagonistic activity at hMOR *in vitro*. The finding also contrasted with Takayama et al. (2002), who found that speciociliatine had negligible opioid agonistic activity in the guinea pig ileum model. The discrepancies could be due to the different types of assays used to assess the functional effect of speciociliatine. Nonetheless, based on both *in vitro* and *in vivo* functional assays, it is possible to hypothesize that the *R* orientation at the C-3 position of speciociliatine enhances its interaction with hMOR, resulting in improved antinociceptive activity compared to mitragynine.

Although indole-based kratom alkaloids have received a lot of attention, little is known about the binding affinity and functional activity of minor oxindole alkaloids. A recent study by Chear et al. (2021) showed that the oxindole alkaloids i.e., corynoxine, corynoxine B, mitragynine oxindole B, and isospeciofoline were predominantly bound to hMOR (K_i_ < 2 μM) compared to hKOR and hDOR (K_i_ > 10 μM). At hMOR, corynoxine exhibited the highest binding affinity with a K_i_ value of 16.4 nM, which is approximately 5 times greater than its C-7 stereoisomer, corynoxine B (K_i_ = 109.8 nM). On the other hand, mitragynine oxindole B and isospeciofoline were moderately bound to hMOR indicating the substitution at the C-9 position of corynoxine/corynoxine B reduces the affinity to hMOR (K_i_ > 1,000 nM). The *in vivo* functional effect of corynoxine was then evaluated using the hot-plate test in rats. The results showed that corynoxine dose-dependently increased antinociception with an ED_50_ value of 6.72 mg/kg which is more potent than morphine (ED_50_ = 12.1 mg/kg). The antinociception of both corynoxine and morphine was also reversed by naltrexone (0.1 mg/kg, i.v.), suggesting that the compounds act as μ-opioid receptor agonists. Interestingly, corynantheidine, an indole precursor of corynoxine/corynoxine B, was discovered to be a functional μ-opioid receptor antagonist (Takayama et al., 2002). The oxidative rearrangement of the indole B-ring caused the shift in μ-opioid antagonistic to agonistic activity. Overall, the above findings suggest that indole and oxindole-based kratom alkaloids could be useful leads for developing new analgesics with fewer side effects that are not derived from morphinan analgesics.

### 3.2 Adrenergic Receptors

In addition to opioid receptors, the adrenergic neurotransmitter system is another major pharmacological target of kratom in treating pain and opioid withdrawal symptoms. Mitragynine was the first kratom alkaloid proven to exert antinociceptive action in rodents via activation of the central adrenergic system. In the hot-plate test, pretreatment with idazoxan (10 μg) was able to reverse the antinociceptive action of mitragynine (10 μg, i.c.v.) in mice (Matsumoto et al., 1996b). Yohimbine (alpha-2 adrenoreceptor antagonist) and prazosin (alpha-1 adrenoreceptor antagonist) also totally and partially suppressed mitragynine antinociceptive activity in a chemotherapy-induced neuropathic pain rat model respectively (Foss et al., 2020). However, information on the specific binding of mitragynine or other kratom alkaloids to various subtypes of alpha-1 and alpha-2 adrenergic receptors is still lacking. As a result, the potential radioligand binding affinities of mitragynine and other indole-based kratom alkaloids i.e. speciogynine, 7-hydroxymitragynine, speciociliatine, corynantheidine, ajmalicine, and tetrahydroalstonine at alpha-1A, 1B, and 1D, and alpha-2A, 2B, and 2C adrenergic receptors were investigated using a high throughput screening approach (Ellis et al., 2020; Obeng et al., 2020). Mitragynine was found to have moderate and non-selective binding affinities at alpha-1A, 1B, and 1D, and alpha-2A, 2B, and 2C, with K_i_ values in the low micromolar range (1.3–9.29 μM). Corynantheidine exhibited high and selective binding affinity at alpha-1D but not alpha-2 adrenergic receptors, with a K_i_ value of 41.7 nM, which is comparable to prazosin, a selective alpha-1D blocker (K_i_ = 0.17 nM) (Obeng et al., 2020). Interestingly, the binding affinity of both mitragynine diastereoisomers i.e. speciociliatine and speciogynine varied at the alpha-2 subtypes. Speciogynine displayed non-selective binding affinities for alpha-2A, 2B, and 2C adrenergic receptors, with K_i_ values ranging from 0.36 to 2.6 μM, similar to its diastereoisomer at the C-20 (mitragynine). Speciociliatine on the other hand was discovered to be less active (K_i_ > 10 μM), implying that the *S*-orientation at the C-3 of mitragynine (speciogynine) is required for binding to alpha-2A, 2B, and 2C adrenergic receptors.

Unlike mitragynine, 7-hydroxymitragynine had little to no binding affinity to both alpha-1 and alpha-2 adrenoreceptors indicating that oxidation at the C-7 abolishes the interaction with these receptors. Both pentacyclic kratom alkaloids i.e. ajmalicine and tetrahydroalstonine showed higher binding affinities on alpha-2A, 2B, and 2C receptors, with K_i_ values in the submicromolar range (K_i_ = 18–65 nM) than tetracyclic kratom alkaloids (K_i_ values in the micromolar range). This shows that, like yohimbine (a potent but non-selective alpha-2 adrenergic antagonist with K_i_ values <5 nM), the ring-D of ajmalicine and tetrahydroalstonine is a critical characteristic for displaying binding to alpha-2A, 2B, and 2C adrenergic receptors (Obeng et al., 2020). The major kratom alkaloids such as mitragynine and speciogynine showed significant binding affinities at alpha-2A, 2B, and 2C adrenergic receptors, which could contribute to kratom overall antinociceptive effect. However, additional research is needed to determine whether the alkaloids work as agonists or antagonists on human adrenergic receptors.

### 3.3 Serotonin Receptors

Serotonin (5-HT) receptors are a class of G-protein-coupled receptors (GPCRs) and ligand-gated ion channels that regulate physiological functions including mood, cognition, sleep, sociability, blood pressure, body temperature, and sexual behavior, through their natural ligand serotonin (Hoyer et al., 1994; Beliveau et al., 2017). 5-HT receptors are known to have at least 14 subtypes from seven distinct families, 5-HT1–5-HT7 (Nichols and Nichols, 2008). Kratom has long been used as a mood enhancer, mild stimulant, or aphrodisiac in traditional settings in Malaysia and Thailand (Singh et al., 2019; Singh et al., 2020b). However, research into its potential interaction with the human serotonin neurotransmission system is still in its early stages.

Matsumoto et al. (1997) reported that mitragynine has a suppressive effect on the central serotonin neurotransmission system. In rodents, pretreatment with mitragynine (i.p.) or ritaserin (i.p.) significantly suppressed the 5-HT2A agonist (5-methoxy-N,N-dimethyltryptamine)-induced head twitch response. The results showed that mitragynine, like ritaserin, acts as a competitive antagonist, blocking the stimulation of the 5-HT2A receptor. Further, mitragynine and its diastereoisomer speciogynine measured K_i_ at 5-HT2A receptor were 7.3 and 2.9 µM respectively in a radioligand binding assay against [^3^H]clozapine (Ellis et al., 2020). In the same study, Ellis et al. also evaluated the 5-HT2A binding affinity of other kratom alkaloids including 7-hydroxymitragynine, corynoxine B, isorhynchophylline, tetrahydroalstonine, and ajmalicine. However, the alkaloids were found to weakly inhibit binding of the radioligand [^3^H]clozapine with K_i_ values >10 μM except for tetrahydroalstonine (K_i_ = 2.6 μM).

Along with the 5-HT2A receptor, indole-based kratom alkaloids such as mitragynine, speciogynine, speciociliatine, and paynantheine have been shown to interact with the 5-HT1A receptor (Obeng et al., 2021). Using *in vitro* displacement of [^3^H]8-OH-DPAT, paynantheine was found to have the highest binding affinity at the human 5-HT1A receptor, with a K_i_ value of 32 nM, followed by speciogynine (39 nM), mitragynine (>1,000 nM), and speciociliatine (>1,000 nM). The *in vivo* binding functionality of the alkaloids at the 5-HT1A receptor was further evaluated by induction of lower lip retraction (LLR) in rats (i.p.) in reference to ipsapirone, a selective 5-HT1A partial agonist. Among the tested alkaloids, speciogynine induced the strongest LLR effect with an ED_50_ value of 23 mol/kg, followed by paynantheine (26 mol/kg) and mitragynine (62 mol/kg). However, the effects were weaker than ipsapirone (ED_50_ = 1.1 mol/kg). The LLR effects of the alkaloids and ipsapirone were reversed by the 5-HT1A receptor antagonist WAY100635 (0.019 μmol/kg, i.p.), suggesting that the alkaloids potentially act as 5-HT1A agonists or partial agonists, in a similar way to ipsapirone. Based on the *in vitro* and *in vivo* findings, it can be assumed that the *R* orientation at C-20 of speciogynine and paynantheine is critical for 5-HT1A agonistic activity. The binding affinity of the alkaloids for the 5-HT1A receptor is dramatically reduced when their orientation is switched from *R* to *S* (mitragynine/speciociliatine). Taking all of this into account, it is hypothesized that the traditional use of kratom as a mood enhancer is due in part to the interaction of its indole-based alkaloids with the 5-HT1A and 5-HT2A receptors.

### 3.4 Dopamine Receptors

The level of dopamine neurotransmitter in the brain is primarily regulated by a group of GPCRs known as dopamine receptors (Beaulieu and Gainetdinov, 2011). There are a total of 5 dopamine receptor subtypes i.e., D1, D2, D3, D4, and D5 regulating emotion, locomotion, memory and learning, sleep, decision making, and the reward system in the human brain (Mishra et al., 2018). Several studies have suggested that dopaminergic receptors are involved in the antipsychotic, antidepressant, anxiolytic, and anti-addiction activities of kratom or its main alkaloid, mitragynine.

Boyer et al. (2008) were the first to report the binding potential of mitragynine to dopamine receptors, specifically the D2 subtype. In the study, mitragynine was found to moderately inhibit the radioligand binding to the D2 receptor, with a percentage inhibition of 54.22%. The *in vitro* finding was supported by several *in vivo* studies utilizing approaches such as elevated plus-maze, apomorphine-induced climbing behavior, haloperidol-induced catalepsy, and ketamine-induced social withdrawal in rodents. Hazim et al. (2014) investigated the potential role of the dopaminergic system in the anxiolytic-like activity of mitragynine in the elevated plus-maze test. The findings showed that a single oral administration of mitragynine (40 mg/kg) increased the percentage of open arm entries and the time spent on open arms, in a similar way to apomorphine, a non-selective dopamine agonist. The effects were significant but not fully antagonized by sulpiride and SCH 23390. Sulpiride is a non-selective D2-like antagonist while SCH 23390 is a selective D1 antagonist (Holanda et al., 2019). These observations suggest that mitragynine is a moderate dopamine agonist, and its anxiolytic-like activity was partly mediated by D1 and D2-like receptors. However, the findings are in contradiction to the findings reported by Vijeepallam et al. (2016) where they discovered that kratom leaf extract exhibited an antipsychotic-like effect in mice through the blockage of the central D2 receptor. Vijeepallam et al. (2016) found that pretreatment with kratom leaf extract (75 and 100 mg/kg, p.o.) significantly reversed apomorphine-induced cage climbing behavior, ketamine-induced hyperactivity, and social withdrawal deficit in mice. Moreover, co-treatment with the leaf extract significantly enhanced the haloperidol-induced catalepsy in mice. Haloperidol is an antipsychotic that acts as a dopamine D2 receptor antagonist. The antidopaminergic action of the leaf extract (1–100 μg/ml) was further assessed in an *ex-vivo* study using isolated rat vas deferens preparation, and the results showed that the extract inhibited the contractility evoked by dopamine in a dose-dependent manner. However, their pEC_50_ values (pEC_50_ 1.01–1.40 μg/ml) were not significantly altered at different treatment doses (1–20 μg/ml) similar to what observed in the treatment with haloperidol (1.6–12.8 μg/ml) (pEC50 1.31–1.53 μg/ml). These results affirm kratom leaf extract acts as a dopamine D2 blocker/antagonist, similar to haloperidol. However, Vijeepallam’s findings are in contradiction with what was reported by Hazim et al. (2014) of which mitragynine acts as a dopamine D1 or D2 agonist, and this could be caused by several factors: 1) mitragynine as a pure compound has a narrow receptor binding profile compared to kratom extract; 2) kratom leaf extract contains multicomponent which might interact with a broad range of CNS receptors leading to the differences in the observed effect; 3) dose-dependent presynaptic (functional antagonistic) and postsynaptic (agonistic) action of kratom and mitragynine at dopamine receptors. Therefore, more research is needed to determine the specific binding profile of mitragynine and other kratom alkaloids at dopamine receptors using *in vitro* radioligand binding assays.

Summary of interactions of kratom alkaloids with CNS receptors is tabulated in Table 3.

**TABLE 3:** Radioligand binding and functional profiles of selected kratom alkaloids.

References	Membrane source	Receptor	Radioligand	Alkaloids	Key findings	Binding affinity	Functional
Takayama et al. (2002)	Guinea pig (rodent)	μ-opioid	[^3^H]DAMGO	Mitragynine, speciociliatine, 7-hydroxymitragynine, mitragynine pseudoindoxyl, corynantheidine, mitragynine n-oxide	Mitragynine, 7-hydroxymitragynine, and mitragynine pseudoindoxyl act as agonists at μ-opioid receptor	Yes	Yes (*In vivo*)
					7-hydroxymitragynine and mitragynine pseudoindoxyl are more potent than morphine		
					Corynantheidine is a selective and functional μ-opioid antagonist		
Kruegel et al. (2016)	Transfected cells (human and rodent)	μ-opioid	[^3^H]DAMGO	Mitragynine, 7-hydroxymitragynine, speciociliatine, paynantheine, speciogynine	7-hydroxymitragynine and mitragynine are partial agonists at human μ-opioid receptor and competitive antagonists at human κ-receptor	Yes	Yes (*In vitro*)
		κ-opioid	[^3^H]U69593		Paynantheine, speciogynine and speciociliatine are competitive antagonists at both human κ- and μ-opioid receptor subtypes		
		δ-opioid	[^3^H]DADLE		Except for 7-hydroxymitragynine and mitragynine, other kratom alkaloids show no notable agonistic or antagonistic effects at rodent opioid receptors. Mitragynine acts as a competitive antagonist at rodent μ-opioid receptor, 7-hydroxymitragynine remains as partial agonist		
Obeng et al. (2020)	Transfected cells (human)	μ-opioid	[^3^H]DAMGO	Mitragynine, Speciociliatine, corynantheidine, 7-hydroxymitragynine	7-hydroxymitragynine is a full agonist at μ-opioid receptor and a competitive antagonist at κ- and δ-opioid receptors	Yes	Yes (*In vivo*) (*In vitro*)
		κ-opioid	[^3^H]U69593		Mitragynine and speciociliatine are partial agonists at μ-opioid receptor. Speciociliatine (K_i_ 54.6 nM; EC_50_ 39.2 nM) is a stronger partial agonist than mitragynine (K_i_ 161 nM; EC_50_ 307.5 nM)		
		δ-opioid	[^3^H]DADLE		Corynantheidine binds selectively to μ-opioid receptor (K_i_ 118 nM)		
Ellis et al. (2020)	Transfected cells (human)	μ-opioid	[^3^H]DAMGO	Mitragynine, speciogynine, ajmalicine, tetrahydroalstonine, corynoxine B, isorhynchophylline	Mitragynine and speciogynine bind to μ- and κ- opioid receptors at low micromolar range (K_i_ 0.74–3.6 μM)	Yes	No
		κ-opioid	[^3^H]U69593		7-hydroxymitragynine shows non-selective and greatest binding affinity to all opioid subtypes (K_i_ < 1 μM)		
		δ-opioid	[^3^H]DADLE		Ajmalicine shows weak or no binding affinity to all opioid receptor subtypes (K_i_ ≥ 10 μM). Corynoxine B and isorhynchophylline bind selectively to μ-opioid receptor with K_i_ 1.6 and 0.54 μM, respectively		
Chear et al. (2021)	Transfected cells (human)	μ-opioid κ-opioid δ-opioid	[^3^H]DAMGO [^3^H]U69593 [^3^H]DADLE	Corynoxine, corynoxine B, isospeciofoline, mitragynine oxindole B, Speciociliatine n-oxide	Corynoxine and corynoxine B exhibit strong and selective binding affinity to μ-opioid receptor with K_i_ 16.4 and 109.8 nM, respectively	Yes	Yes (*In vivo*)
					Corynoxine acts as a μ-opioid receptor agonist in hot-plate test (10 mg/kg) and the effect is reversed by naltrexone		
			
Obeng et al. (2020)	Transfected cells (human)	Alpha-1A	[^3^H]prazosin	Mitragynine, speciociliatine, corynantheidine, 7-hydroxymitragynine	Mitragynine binds to alpha-1 and alpha-2 subtypes (K_i_ at low micromolar range). Mitragynine is a partial agonist at alpha-1A,D, but acts as a competitive antagonist at alpha-1B,2C	Yes	Yes (*In vitro*)
		Alpha-1B	[^3^H]prazosin				
		Alpha-1D	[^3^H]prazosin				
		Alpha-2A	[^3^H]RX821002		Corynantheidine binds to alpha-1D receptor (K_i_ 41.7 nM)		
		Alpha-2B	[^3^H]RX821002				
		Alpha-2C	[^3^H]RX821002				
Ellis et al. (2020)	Transfected cells (human)	Alpha-2A	[^3^H]rauwolscine	Mitragynine, speciogynine, 7-hydroxymitragynine, ajmalicine, corynoxine B, isorhynchophylline	Mitragynine and speciogynine show non-selective binding affinity to all subtypes at low micromolar range (K_i_ 0.36–4.9 μM)	Yes	No
		Alpha-2B			Oxygenated or oxindole alkaloids K_i_ > 10 μM for adrenergic receptors (not active)		
		Alpha-2C			Ajmalicine exhibits non-selective binding affinity to all alpha-2 subtypes (K_i_ 18–65 nM)		
Ellis et al. (2020)	Transfected cells (human)	5-HT1A	[^3^H]8-OH-DPAT	Mitragynine, speciogynine, ajmalicine, tetrahydroalstonine, corynoxine B, isorhynchophylline	Mitragynine and speciogynine K_i_ 0.54–7.3 μM	Yes	No
		5-HT2A	[^3^H]clozapine		Ajmalicine and tetrahydroalstonine 5-HT1A K_i_ < 0.5 μM. Oxygenated indole and oxindole alkaloids		
Obeng et al. (2021)	Transfected cells (human)	5-HT1A	[^3^H]8-OH-DPAT	Mitragynine, paynantheine, speciogynine, speciociliatine	Binding affinity: paynantheine (32 nM) > speciogynine (39 nM) > mitragynine (>1,000 nM) and speciociliatine (>1,000 nM)	Yes	Yes (*In vivo*)
					Speciogynine, paynantheine and mitragynine are 5-HT1A agonists		
Boyer et al. (2008)	Not specified	μ-opioid; κ-opioid; δ-opioid; Alpha-2; D2; 5-HT2C; 5-HT7; A2A	Not specified	Mitragynine	Mitragynine binds to μ and κ-opioid receptors (∼90% inhibition) but not δ-opioid receptor	No	No
						(% inhibition of radioligand binding at single dose screening)	

## 4 Interactions of Kratom Alkaloids With Cellular Barriers

Cellular barriers formed by epithelium that lined tissue cavities and endothelium that lined blood vessels delineate tissue compartments and play a pivotal role in maintaining homeostasis, and protecting the tissue microenvironment. The barriers function as a gatekeeper, regulating the passage of substances across the tissue compartments through restrictive tight junctions between adjacent cells; and concerted action of transporters that transport essential nutrients required by the tissues, and keeping out xenobiotics and other harmful substances (Abbott et al., 2010; Vancamelbeke and Vermeire, 2018). In drug discovery and development, it is acknowledged that the barriers imposed a significant hurdle due to the restrictive nature of the barriers which would limit successful delivery of therapeutic molecules to the site of action. It is also known that the functions of the barriers are altered in pathophysiology (Chelakkot et al., 2018; Sweeney et al., 2019). Here, interactions of kratom alkaloids with cellular barriers are discussed within the scope of barrier permeability of the alkaloids, involvement with transporters expressed at the barriers, and effects of the alkaloids on the barrier function.

### 4.1 Barrier Permeability

The most widely used method to measure barrier permeability is by utilizing two-dimensional *in vitro* cell-based models. The models are established by culturing epithelial or endothelial cells on semi-permeable membrane of well-plate inserts to yield confluent cell monolayers. Determination of barrier properties of the cells particularly tight junction tightness and functional expression of polarized membrane transporters are carried out to evaluate the goodness of purpose of the models. Following the model validation, *in vitro* permeability assay of a compound of interest is conducted. Quantitative analysis of the compound present in assay buffer sampled from the apical and the basolateral compartments which are separated by the cell monolayer enables determination of apparent permeability coefficient, *P*_app_ of the compound. Comparison of the *P*_app_ with *P*_app_ of reference drug would give insights to the potential of barrier permeation of the compound.

For the intestinal barrier, the Caco-2 cell line developed from human colorectal adenocarcinoma epithelium is commonly used to establish a model for the barrier, to determine intestinal absorption (Volpe, 2020). The model was used to investigate intestinal permeability of kratom alkaloids mitragynine (Manda et al., 2014; Rusli et al., 2019), 7-hydroxymitragynine, and mitraphylline (Manda et al., 2014). Mitragynine was found to be the most permeable across the Caco-2 cells, followed by 7-hydroxymitragynine and mitraphylline with *P*_app_ of 24.2 × 10^–6^ cm/s, 16.1 × 10^–6^ cm/s and 6.3 × 10^–6^ cm/s respectively, when tested at 5 μM in the absorptive direction (apical to basolateral). *P*_app_ values in the absorptive direction for the three compounds were similar when tested at 10 μM (Manda et al., 2014). Rusli et al. (2019) reported comparable mitragynine *P*_app_ of 18.8 × 10^–6^ cm/s. The permeability of mitragynine across the intestinal barrier was also measured using *in situ* single-pass perfusion technique in rats (Jagabalan et al., 2019). *In situ* perfusion technique enables measurement of barrier permeability in an intact functional barrier with membrane transporter machinery in place (Jeong et al., 2004). The findings showed that mitragynine *P*_eff_ was 111 × 10^–6^ cm/s. Manda et al. (2014) and Jagabalan et al. (2019) both included high permeability reference drug i.e. propranolol in their studies. Mitragynine showed comparable permeability coefficients to the drug where *P*_app_ of 24.2 × 10^–6^ cm/s (5 μM) and 25.3 × 10^–6^ cm/s (10 μM) were measured using the *in vitro* Caco-2 model while propranolol showed *P*_app_ of 34.2 × 10^–6^ cm/s (Manda et al., 2014); *P*_eff_ of 111 × 10^–6^ cm/s was measured using the *in situ* technique while propranolol showed *P*_eff_ of 127 × 10^–6^ cm/s (Jagabalan et al., 2019).

Previous studies on the BBB permeability of kratom alkaloids utilized *in vitro* models from epithelial and endothelial cells (Manda et al., 2014; Yusof et al., 2019). Compared to endothelial cells, epithelial cell monolayer more readily shows restrictive tight junctions which is the hallmark of the BBB. However, use of primary brain endothelial cells or differentiated stem cells, and co-culture of endothelial cells with other cells of the neurovascular unit for example astrocytes could contribute to having endothelial cell monolayer with restrictive tight junctions and close phenotypic resemblance to the BBB *in vivo* (see Helms et al., 2016 for the different *in vitro* BBB models available). In the MDR-MDCK epithelial cell model, mitragynine and 7-hydroxymitragynine showed apical to basolateral, or blood to brain side *P*_app_ of 15.3 × 10^–6^ cm/s and 12.4 × 10^–6^ cm/s when tested at 5 μM; 16.2 × 10^–6^ cm/s and 13.2 × 10^–6^ cm/s when tested at 10 μM respectively (Manda et al., 2014). When the two alkaloids were assayed using primary porcine brain endothelial cells, mitragynine showed apical to basolateral *P*_app_ of 31.8 × 10^–6^ cm/s, while 7-hydroxymitragynine *P*_app_ was 15.3 × 10^–6^ cm/s (Yusof et al., 2019). Based on the two studies, mitragynine showed approximately 1.2–2.1 times higher BBB permeability than 7-hydroxymitragynine. This could potentially be contributed by differences in physicochemical properties of the alkaloids. Mitragynine being more lipophilic and 7-hydroxymitragynine being more polar might eased and hampered passive transcellular permeation respectively. Another possibility is the involvement of membrane transporters to transport the alkaloids. Meanwhile, mitraphylline apical to basolateral permeability was more restricted with *P*_app_ of 3.3 × 10^–6^ cm/s when tested at 5 μM, and 3.4 × 10^–6^ cm/s when tested at 10 μM (Manda et al., 2014).

### 4.2 Interactions With Membrane Transporters

Physiological barriers not only act as a physical barrier which is contributed by the restrictive tight junctions, but also as metabolic and transport barriers to permeation of molecules (Abbott et al., 2006). The transport barrier is imposed by solute carrier (SLC) and ATP-binding cassette (ABC) transporters, which function to either facilitate or impede transcellular permeability across the barriers. In drug discovery and development, prediction or determination of compounds’ potential substrates for the ABC transporters particularly the P-glycoprotein (P-gp) is often considered. Efflux by the P-gp which has broad substrate specificity could affect the pharmacokinetics of a compound such as limiting intestinal absorption, impeding CNS penetration, and thus influencing drug delivery and targeting (Lin and Yamazaki, 2003; Miller, 2015). To overcome this, modulation of the P-gp function to reduce efflux, or alteration of the P-gp expression are some of the approaches being explored to improve drug delivery (Miller, 2015).

Evidence on mitragynine and 7-hydroxymitragynine P-gp-mediated efflux are conflicting (Table 4). Lack of polarization in bidirectional transport measured *in vitro* indicated no potential efflux, and unaltered permeability in presence of P-gp inhibitors suggested that the alkaloids were not substrates of P-gp (Manda et al., 2014; Meyer et al., 2015; Jagabalan et al., 2019; Rusli et al., 2019). However, when the alkaloids were tested at submicromolar concentration, an increase in apical to basolateral permeability was observed in presence of the P-gp inhibitor, valspodar (PSC833), suggesting P-gp-mediated efflux (Yusof et al., 2019). The differences in concentrations used to test the alkaloids in the studies may explain the discrepancies of the findings, as higher concentrations can cause transporter saturation, and this, in turn, affects readouts of *in vitro* bidirectional permeability assay (Saaby and Brodin, 2017). On the other hand, P-gp-mediated efflux of mitraphylline was evident from Manda et al. study.

**TABLE 4:** Functional interactions of kratom alkaloids with efflux transporters.

Alkaloid	Concentration tested	Methods	Findings	Subjected to efflux	Efflux transporter inhibition	References
Mitragynine	5, 10 μM	*In vitro* bidirectional permeability assay using Caco-2 and MDR-MDCKII cells	No polarization of transport. Efflux ratio = 1.0 and 1.1	No	—	Manda et al. (2014)
	5 μM	*In vitro* permeability assay using Caco-2 cells with or without P-gp inhibitor verapamil (5 μM)	Permeability was unaltered in presence of verapamil	No	—	Meyer et al. (2015)
	10 μM	*In vitro* bidirectional permeability assay using Caco-2 cells	No polarization of transport. Efflux ratio = 0.9	No	—	Rusli et al. (2019)
	0.3 μM	*In vitro* permeability assay using primary porcine brain endothelial cells with or without P-gp inhibitor valspodar (PSC833; 1 μM)	Increased apical to basal permeability (blood to brain side) in presence of valspodar	Yes (P-gp)	—	Yusof et al. (2019)
		Brain extent study—combinatory approach of *in vivo* neuropharmacokinetic, *in vitro* drug tissue binding and brain slice assays	*K*_p,uu,brain_ < 1 indicating net efflux	Yes		
	40 μg/ml	*In situ* single pass intestinal perfusion in small intestine of rats with or without P-gp inhibitor azithromycin (200 μg/ml)	Permeability was unaltered in presence of azithromycin	No	—	Jabagabalan et al. (2019)
	—	*In vitro* uptake assay of P-gp substrate calcein-AM in presence of mitragynine at different concentrations	Increased uptake of calcein-AM in MDR-MDCKII cells in presence of mitragynine dose-dependently (EC_50_ = 18.2 μM)	—	Yes (P-gp)	Manda et al. (2014)
	5 μM	*In vitro* permeability assay of P-gp substrate rhodamine 123 across Caco-2 cell monolayers with or without mitragynine in basolateral to apical (secretory) direction	Reduced basolateral to apical permeability of rhodamine 123 in presence of mitragynine	—	Yes (P-gp)	Meyer et al. (2015)
	10 μM	*In vitro* permeability assay of P-gp substrate digoxin across Caco-2 cell monolayers with or without mitragynine	Reduced basolateral to apical permeability of digoxin in presence of mitragynine	—	Yes (P-gp)	Rusli et al. (2019)
	0.3 μM	*In vitro* permeability assay of P-gp substrate digoxin across primary porcine brain endothelial cell monolayers with or without mitragynine in apical to basolateral (absorptive) direction	Increased apical to basolateral permeability of digoxin in presence of mitragynine	—	Yes (P-gp)	Yusof et al. (2019)
	5, 50, 500 μM	Human BCRP (hBCRP) ATPase activity	Mitragynine stimulated hBCRP ATPase at all concentrations tested, and inihibited hBCRP ATPase at 500 μM	Yes	Possibly weak inhibition due to IC_50_ value	Wagmann et al. (2018)
	5–2,500 μM	Determination of IC_50_	IC_50_ = 359 μM			
7-hydroxy-mitragynine	5, 10 μM	*In vitro* bidirectional permeability assay using Caco-2 and MDR-MDCKII cells	No polarization of transport. Efflux ratio = 1.2	No	—	Manda et al. (2014)
	0.3 μM	*In vitro* bidirectional permeability assay using primary porcine brain endothelial cells	Higher basolateral to apical (brain to blood side) permeability. Efflux ratio = 1.39	Yes (P-gp)	—	Yusof et al. (2019)
	0.3 μM	*In vitro* permeability assay with or without P-gp inhibitor valspodar (PSC833; 1 μM)	Increased apical to basolateral permeability (blood to brain side) in presence of valspodar			
	—	Brain extent study—combinatory approach of *in vivo* neuropharmacokinetic, *in vitro* drug tissue binding and brain slice assays	*K*_p,uu,brain_ < 1 indicating net efflux	Yes		
	—	*In vitro* uptake assay of P-gp substrate calcein-AM in presence of 7-hydroxymitragynine at different concentrations	Increased uptake of calcein-AM in MDR-MDCKII cells in presence of 7-hydroxymitragynine dose-dependently (EC_50_ = 32.4 μM)	—	Yes (P-gp)	Manda et al. (2014)
	0.3 μM	*In vitro* permeability assay of P-gp substrate digoxin across primary porcine brain endothelial cell monolayers with or without 7-hydroxymitragynine in apical to basolateral (absorptive) direction	Increased apical to basolateral permeability of digoxin in presence of 7-hydroxymitragynine	—	Yes (P-gp)	Yusof et al. (2019)
Mitraphylline	5, 10 μM	*In vitro* bidirectional permeability assay using Caco-2 and MDR-MDCKII cells	Higher basolateral to apical (secretory) permeability with efflux ratio of 3.3–6.6	Yes		Manda et al. (2014)
	—	*In vitro* uptake assay of P-gp substrate calcein-AM in presence of mitraphylline at different concentrations	No effect on calcein-AM uptake		No	Manda et al. (2014)

Previous studies are in agreement that mitragynine and 7-hydroxymitragynine inhibited P-gp-mediated efflux of known substrates of the transporter (Table 4). The alkaloids dose-dependently increased MDR-MDCK cell uptake of calcein-AM, with mitragynine and 7-hydroxymitragynine EC_50_ of 18.2 and 32.4 μM respectively, comparable to the P-gp inhibitor verapamil which shown EC_50_ of 22.3 μM (Manda et al., 2014). In the Caco-2 model, mitragynine was demonstrated to reduce the permeability of rhodamine 123 and digoxin in the basolateral to the apical direction (secretory direction) at 5 and 10 μM (Meyer et al., 2015; Rusli et al., 2019). We also found evidence for mitragynine and 7-hydroxymitragynine inhibition of P-gp-mediated efflux of digoxin at a concentration of 0.3 μM, comparable to inhibition by valspodar (Yusof et al., 2019). The inhibition of P-gp-mediated efflux by kratom alkaloids needs careful considerations as this could potentially cause interactions with drugs that are substrates of the P-gp. Co-presence of the alkaloids and the drugs may lead to an increase in the drugs absorption and tissue distribution, and decrease elimination. While this could be a strategy for the drugs to reach sites of action, the non-specific inhibition of the P-gp in non-targeted tissues could contribute to cytotoxicity.

Another important ABC transporter which expression includes at the gastrointestinal tract and at the BBB is the breast cancer resistance protein (BCRP). At the human BBB, the BCRP expression was found to be the most abundant among the ABC transporters, 1.34 fold higher than the P-gp expression; while the opposite was found for mice where the P-gp expression was 3.20 fold higher than the BCRP expression (Uchida et al., 2011). This need to be taken into consideration when extrapolating data from mice to human. The two transporters have been reported to work cooperatively in limiting the entry of chemotherapeutic drugs into the brain, and inhibition of one transporter can be compensated by the other (Agarwal et al., 2011). Based on human BCRP ATPase activity where the formation of ADP was quantified as an indicator for either stimulation or inhibition of the transporter in presence of test compounds, mitragynine was reported as a potential substrate of the BCRP and could inhibit the transporter function with an IC_50_ value of 359 μM (Wagmann et al., 2018). Findings reported by Wagmann et al. (2018) and Yusof et al. (2019) provide evidence for mitragynine dual substrate of the P-gp and the BCRP.

Efflux of mitragynine and 7-hydroxymitragynine was also determined in the study of the alkaloids extent in the brain (Yusof et al., 2019). By using a combinatory approach of *in vivo* neuropharmacokinetic, *in vitro* drug tissue binding and brain slice assays to calculate total whole brain to plasma concentration ratio (*K*_p,brain_), fraction of unbound alkaloids in plasma (*f*_u,plasma_), and volume of distribution of unbound alkaloids in the brain (*V*_u,brain_) respectively, the extent of unbound alkaloids in the brain (*K*_p,uu,brain_) yielded values of approximately 0.1, which is below the value of unity (1), thus indicating efficient efflux of the alkaloids (Yusof et al., 2019).

Apart from interactions with the efflux transporters, mitragynine could also potentially be transported by influx transporters into the brain (Yusof et al., 2019). However, further investigations are needed to confirm this.

### 4.3 Alteration of Barrier Function

*In vitro* cell-based models of physiological barriers not only are great tools to investigate mechanisms of permeability but can also be used to determine the effects of exposure to compounds on the structure and function of the barriers. Exposure to mitragynine at 40 and 60 μM for 48 h reduced the viability of human aortic endothelial cells, which was linked to an increase in intracellular reactive oxygen species (ROS) production, leading to caspase-3 activation, DNA fragmentation, and apoptosis (Matsunaga et al., 2017). The LC_50_ determined was 43.1 μM. The effect of mitragynine on the tight junction function of the human aortic endothelial cells was also investigated. The cells grown on semi-permeable inserts were exposed to mitragynine at 5 μM either for a short, or long-term incubation of 5 days. The cells were also incubated with 10 and 20 μM mitragynine for 5 days. The transendothelial electrical resistance was then measured as an indicator for tight junction integrity. Tight junction leakage to FITC-dextran with a molecular weight of approximately 150 kDa was assessed. Findings from the study showed that the long-term exposure to mitragynine caused a decrease in tight junction tightness of the human aortic endothelial cells at all concentrations tested i.e. 5, 10, and 20 μM, which might contribute to leakage of the FITC-dextran at 20 μM (Matsunaga et al., 2017). The decrease in tightness of the tight junction was not observed in cells pre-treated with ROS inhibitor, while ROS generators made it worse. This indicates the involvement of ROS in the disruption of tight junction integrity of the human aortic endothelial cells upon exposure to mitragynine (Matsunaga et al., 2017).

Mitragynine was found to alter the expression of the P-gp. The Caco-2 cells incubated with mitragynine at 0.1, 1, and 10 μM for 72 h showed downregulation of mRNA and protein expression of the P-gp in a concentration-dependent manner (Rusli et al., 2019). The downregulation of expression correlates with reduced intensity of P-gp staining of the cells. The number of cells expressing P-gp was also reduced (Rusli et al., 2019).

Evidence of alteration of cellular barrier function by mitragynine in long-term exposure is concerning. In particular when the concentrations that affected the function falls within the range of mitragynine concentrations reported in human plasma, of which a range of 1.13–5.77 μM was reported in a recent study by Vicknasingam et al. (2020). Future studies should look into other potential alterations to the barrier structure and function as part of safety evaluations.

## 5 Conclusion

Here, we have gathered and discussed physiological interactions of kratom alkaloids within the scope of interactions with drug-metabolizing enzymes and potential for drug-drug interactions, interactions with central nervous system receptors to relate with pharmacological actions, and interactions with cellular barriers of which are not limited to mechanisms of barrier permeability, but also effects of exposure to kratom alkaloids on the barrier function. Although the interactions with enzymes and the receptors may not be necessarily new in regards to kratom research, these areas have gained renewed interest among researchers in recent years due to the wealth of evidence on pharmacological actions of the alkaloids in preclinical studies, the rise of kratom use for self-treatment purposes, and the controversies surrounding the consumption of kratom. Meanwhile, interactions of kratom alkaloids with cellular barriers are largely unexplored.

Highlights from the discussion include the potential for clinically relevant drug-drug interaction due to modulation either in expression or function of drug-metabolizing enzymes, particularly the cytochrome P450 enzymes by the alkaloids. Secondly, kratom alkaloids have been known as atypical opioids stem from the discoveries of their opioids and non-opioids mechanistic. This multimechanistic property of the alkaloids could provide interesting avenues for the development of multi-targeted therapeutics for better efficacy and reduced side effects. As traditional uses generally involve consumption of a brewed drink, the mechanistic of the alkaloids as single compounds and in combination need to be delved deeper. Thirdly, cellular barriers imposed formidable hurdles in the development of therapeutics due to their protective nature and dynamic regulation of the tissue microenvironment. Therefore, a good understanding of the alkaloids’ molecular traffic between physiological interfaces will aid future delivery strategies. As kratom alkaloids have been demonstrated to interact with membrane transporters particularly the efflux transporters, this could also imply the potential for drug-drug interaction with the transporter substrates. Taken together, interactions of kratom alkaloids with drug-metabolizing enzymes and cellular barriers not only affect their tissue distributions and the concentrations at target receptor sites to elicit functional responses, but also distributions, and functional responses of other drugs. As always, more work is needed to understand the physiological interactions of kratom alkaloids in the course of further development as potential therapeutics.
